# A multi-country study using MALDI-TOF mass spectrometry for rapid identification of *Burkholderia pseudomallei*

**DOI:** 10.1186/s12866-021-02276-1

**Published:** 2021-07-16

**Authors:** Wanitda Watthanaworawit, Tamalee Roberts, Jill Hopkins, Ian Gassiep, Robert Norton, Matthew T. Robinson, Joy Silisouk, Poda Sar, Sena Sao, Premjit Amornchai, Direk Limmathurotsakul, Vanaporn Wuthiekanun, Francois Nosten, Andrew J. H. Simpson, Paul Turner, Clare L. Ling

**Affiliations:** 1 Shoklo Malaria Research Unit, Mahidol-Oxford Tropical Medicine Research Unit, Faculty of Tropical Medicine, Mahidol University, Mae Sot, Thailand; 2 Lao-Oxford-Mahosot Hospital-Wellcome Trust Research Unit, Microbiology Laboratory, Mahosot Hospital, Vientiane, Lao People’s Democratic Republic; 3 Cambodia Oxford Medical Research Unit, Angkor Hospital for Children, Siem Reap, Cambodia; 4 Centre for Tropical Medicine and Global Health, Nuffield Department of Medicine, University of Oxford, Oxford, UK; 5 University of Queensland Centre for Clinical Research, Herston, Queensland Australia; 6 Pathology, Townsville Hospital, Townsville, Queensland Australia; 7 Faculty of Medicine, University of Queensland, Brisbane, Australia; 8 Mahidol-Oxford Tropical Medicine Research Unit, Faculty of Tropical Medicine, Mahidol University, Bangkok, Thailand

**Keywords:** *Burkholderia pseudomallei*, Melioidosis, *Burkholderia thailandensis*, MALDI-TOF, Vitek MS, Superspectra, Rapid identification, Mass spectrometry

## Abstract

**Background:**

*Burkholderia pseudomallei* is the bacterial causative agent of melioidosis, a difficult disease to diagnose clinically with high mortality if not appropriately treated. Definitive diagnosis requires isolation and identification of the organism. With the increased adoption of MALDI-TOF MS for the identification of bacteria, we established a method for rapid identification of *B. pseudomallei* using the Vitek MS, a system that does not currently have *B. pseudomallei* in its *in-vitro* diagnostic database.

**Results:**

A routine direct spotting method was employed to create spectra and SuperSpectra. An initial *B. pseudomallei* SuperSpectrum was created at Shoklo Malaria Research Unit (SMRU) from 17 reference isolates (46 spectra). When tested, this initial SMRU SuperSpectrum was able to identify 98.2 % (54/55) of Asian isolates, but just 46.7 % (35/75) of Australian isolates. Using spectra (430) from different reference and clinical isolates, two additional SMRU SuperSpectra were created. Using the combination of all SMRU SuperSpectra with seven existing SuperSpectra from Townsville, Australia 119 (100 %) Asian isolates and 31 (100 %) Australian isolates were correctly identified. In addition, no misidentifications were obtained when using these 11 SuperSpectra when tested with 34 isolates of other bacteria including the closely related species *Burkholderia thailandensis* and *Burkholderia cepacia.*

**Conclusions:**

This study has established a method for identification of *B. pseudomallei* using Vitek MS, and highlights the impact of geographical differences between strains for identification using this technique.

**Supplementary Information:**

The online version contains supplementary material available at 10.1186/s12866-021-02276-1.

## Background

*Burkholderia pseudomallei* is an oxidase positive, Gram-negative bacterium found in the environment and is the causative agent of melioidosis [1]. Melioidosis is endemic to tropical regions, in particular South-East (SE) Asia and Northern Australia with a high case fatality if not appropriately treated [2, 3]. The disease is thought to be under-reported in some countries, with 165,000 melioidosis cases and 89,000 deaths being estimated globally in 2015 [4]. A definitive diagnosis of melioidosis is made through isolation and identification of *B. pseudomallei*, which can take up to four days using conventional methods, including growth on selective medium (Ashdown’s agar and broth), biochemical tests (e.g. bioMérieux API 20NE tests), antimicrobial disc susceptibility tests and latex agglutination tests [5–9]. To enable prompt and appropriate treatment, quicker identification methods are required. Although the latex agglutination test is a rapid method, false positives and negatives can occur and results need to be confirmed with an additional method [10, 11]. In addition, local knowledge of the disease and use of specific tests (i.e. selective agar and latex agglutination) is limited in non-endemic areas, which has implications for identification of infections acquired locally and abroad [12].

Matrix assisted laser desorption/ionization time-of-flight mass spectrometry (MALDI-TOF MS) systems are increasingly being used in diagnostic microbiology laboratories. MALDI-TOF MS offers a non-specific and rapid method for bacteria identification. *B. pseudomallei* is not currently part of the *in-vitro* diagnostic (IVD) databases on the two MALDI-TOF MS systems used for bacterial identification in laboratories: the MALDI Biotyper (Bruker Daltonik GmbH) and the Vitek MS (bioMérieux) systems. This means that currently *B. pseudomallei* isolates will be mis- or unidentified using these systems. *B. pseudomallei* is part of the MALDI Biotyper and Vitek MS research use only (RUO) databases. However, only a limited number of strains have been used and these databases are not Food and Drug Administration (FDA) approved or clinically evaluated [13]. There have been several studies demonstrating the utility of the MALDI Biotyper for the identification of *B. pseudomallei* [10, 14–17], but fewer using the Vitek MS. Using the MALDI Biotyper IVD database, isolates have been misidentified as *B. thailandensis* [13], a closely related environmental bacterial species that shares the same geographical distribution as *B. pseudomallei* in SE Asia [7].

There have been two previous studies investigating the use of the Vitek MS for the identification of *B. pseudomallei* [18, 19]. Vitek MS RUO uses a database composed of SuperSpectra, developed from multiple single-isolate spectra, to identify bacteria. A study from Australia used 85 isolates (899 spectra) to create SuperSpectra, which correctly identified 99.8 % of spectra [18]. This study showed that testing isolates in triplicate (three spots per isolate) increased correct identification from 41 % of isolates obtained with one spot to 100 %. Another study from China used 10 strains of *B. pseudomallei* and 10 strains of *B. thailandensis* to make two SuperSpectra [19]. Isolates, 26 *B. pseudomallei* and 80 other *Burkholderia* sp., were then run against these SuperSpectra with a 100 % correct identification rate. Both of these studies only used isolates from their respective countries. Several studies have shown a high rate of genetic diversity in *B. pseudomallei* from different locations with distinct populations being identified from Australia and Asia [20, 21] and even variation within country [22]. It is unknown whether this genetic diversity might have an impact on MALDI-TOF identification using a SuperSpectrum developed from isolates from a limited geographic distribution.

The aim of this study was to create a SuperSpectrum for *B. pseudomallei* identification using the Vitek MS in RUO mode and test it against isolates from three different SE Asian countries where melioidosis is endemic. This SuperSpectrum was then used to identify Australian isolates from a previous study performed at Townsville Hospital, referred to as ‘Townsville’ [18] to determine the accuracy of the newly made SuperSpectrum and its utility for identifying *B. pseudomallei* isolates from other regions.

## Results

### Viability check

Growth was not detected after incubation of the plates for 14 days at any of the three sites.

### SuperSpectra creation and initial testing

**Fig. 1:**
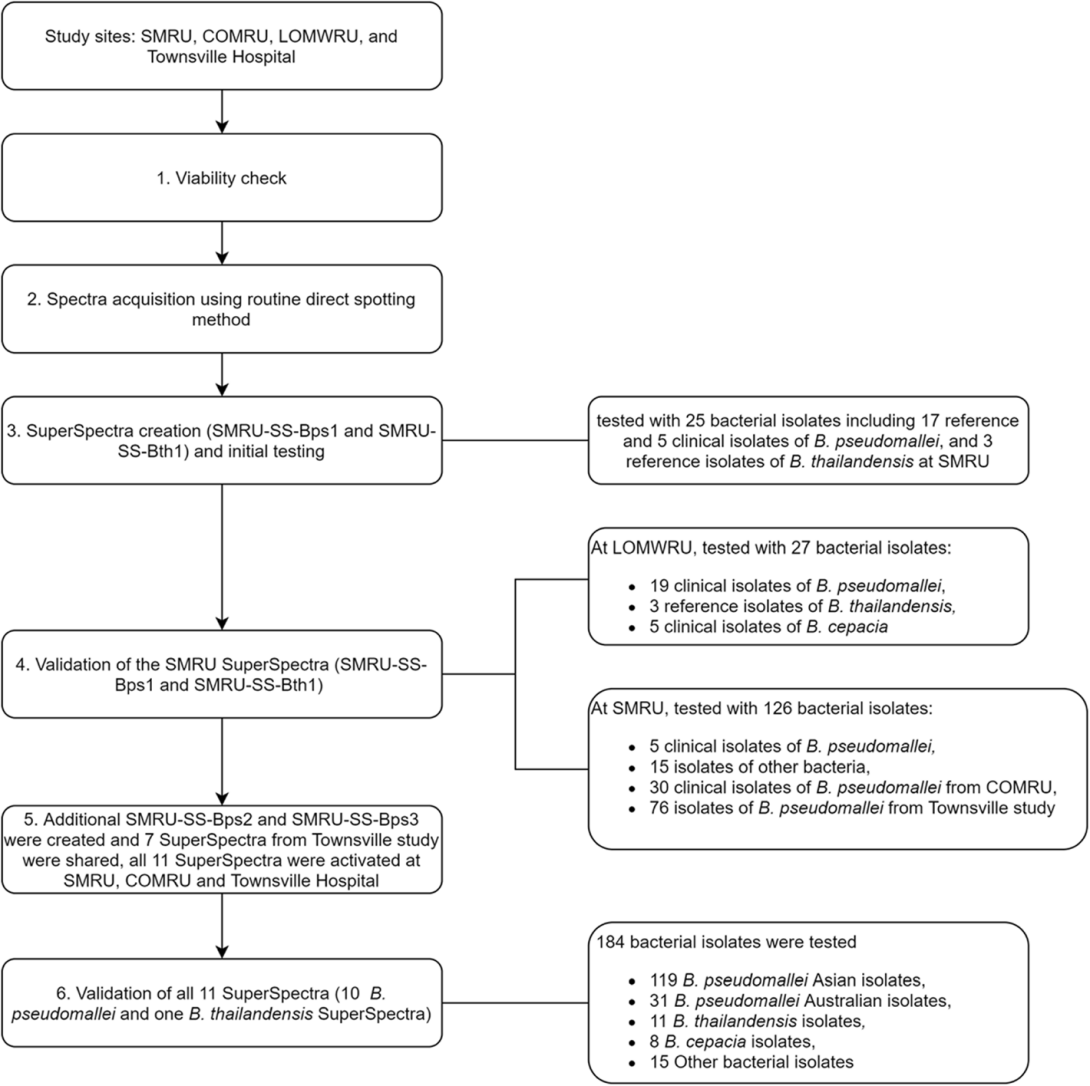
Study flow chart

During initial spectra acquisition, none of 17 reference isolates of *B. pseudomallei* were given an identification prior to the SuperSpectra creation. Once the SuperSpectra SMRU-SS-Bps1 and SMRU-SS-Bth1 were created (Fig. 1, step 3), they were checked against available isolates at SMRU. Correct identification was obtained for 25/25 isolates. To investigate the consistency of detection and quality of spots, all 25 isolates were spotted in triplicate. The results showed that the correct identification was obtained from 3/3 replicates for 19 isolates (76.0 %) (16 *B. pseudomallei* and three *B. thailandensis*), 2/3 replicates for three isolates (12.0 %) and 1/3 replicates for three isolates (12.0 %). Of all 66 identified spectra, 63 spectra had 99.9 % identity. The three spectra that had < 99.9 % identity included two spectra of *B. pseudomallei* (89.1 and 82.5 %) and one spectrum of *B. thailandensis* (95.2 %) (Additional file 1, Fig. S1; 1H1, 2E3 and 2C1 respectively). Spectra that did not provide an identification of *B. pseudomallei*, failed to produce an identification rather than giving a false one. For the nine spots that had no identification, only one spot was bad quality (1I3, Additional file 1, Fig. S1).

### Validation of the SMRU SuperSpectra

Using reference and clinical isolates from the Mahidol-Oxford Tropical Medicine Research Unit (MORU) network laboratories, 53/54 (98.1 %) isolates (146/162 [90.1 %] spectra) were correctly identified as *B. pseudomallei* using the SMRU-SS-Bps1 and SMRU-SS-Bth1 SuperSpectra (Fig. 1, step 4). Although no misidentification of any spectra was observed, 1/54 (1.9 %) isolate (16/162 [9.9 %] spectra) failed to produce an identification. The identification accuracy by study sites are shown in Table 1. To investigate the consistency of detection, all 54 isolates were spotted in triplicate. The results showed that for the 53 isolates that were correctly identified, 40 isolates were detected in 3/3 replicates, eight isolates were detected in 2/3 replicates and five isolates were detected in 1/3 replicates. No misidentifications of *B. pseudomallei* were obtained when tested with 23 non-*B. pseudomallei* isolates (61 spectra).

**Table 1:** Identification accuracy of *B. pseudomallei* SuperSpectra by site

SuperSpectra	Spectrum acquisition site/isolate origin	Correctly identified spectra (%)	Correctly identified isolates (%)	Unidentified spectra (%)	Unidentified isolates (%)	Misidentified spectra (%)	Misidentified isolates (%)
SMRU^a^	SMRU	15/15 (100)	5/5 (100)	0	0	0	0
	COMRU	90/90 (100)	30/30 (100)	0	0	0	0
	LOMWRU	41/57 (71.9)	18/19 (94.7)	16/57 (28.1)	1/19 (5.3)	0	0
	Townsville [18]^c,d^	143/715 (20.0%)	36/76 (47.4)	563/715 (78.7)	33/76 (43.4)	9/715 (1.3)	7/76 (9.2)
	Asia	148/192 (77.1)	54/55 (98.2)	44/182 (22.9)	1/54 (1.9)	0	0
	Australia	136/685 (19.9)	35/75 (46.7)	540/685 (78.8)	33/75 (44.0)	9/685 (1.3)	7/75 (9.3)
SMRU+Townsville^b^	SMRU	14/15 (93.3)	5/5 (100)	1/15 (6.7)	0	0	0
	COMRU	277/285 (97.2)	95/95 (100)	8/285 (2.8)	0	0	0
	LOMWRU	38/57 (66.7)	19/19 (100)	19/57 (33.3)	0	0	0
	Townsville/Australia^e^	59/62 (95.2)	31/31 (100)	2/62 (3.2)	0	1/62 (1.6)^f^	0
	Asia^e^	329/357 (92.2)	119/119 (100)	28/357 (7.8)	0	0	0

^a^SMRU-SS-Bps1 and SMRU-SS-Bth1

^b^SMRU-SS-Bps1-3, SMRU-SS-Bth1, Townsville-SS-Bps128, Townsville-SS-Bps347, Townsville-SS-Bps457, Townsville-SS-Bps694, Townsville-SS-Bps854, Townsville-SS-BpsATCC4846, and Townsville-SS-BpsATCC23343

^c^Seventy-four isolates from Australia, 1 isolate from Asia and 1 isolate of unknown origin

^d^Available existing spectra from the Townsville study were used and bacterial isolates were not re-cultured

^e^Independent isolates that were not used in the SuperSpectra creations

^f^Misidentified as *Sphingobacterium multivorum* (91.5% ID) another spot of the same isolate was correctly identified as *B. pseudomallei* (99.9% ID)

### Identification of Townsville isolates using SMRU SuperSpectra

Using SMRU-SS-Bps1 and SMRU-SS-Bth1, only 36/76 (47.4 %) isolates (143/715 [20.0 %] spectra) from Townsville were correctly identified as *B. pseudomallei*, and 7/76 (9.2 %) isolates (9/715 [1.3 %] spectra) were misidentified as *B. thailandensis* (Table 1).

### Identification of Asian and Australian isolates using SMRU SuperSpectra

With the initial SMRU SuperSpectra, SMRU-SS-Bps1 and SMRU-SS-Bth1, 54/55 (98.2 %) Asian isolates (148/192 [77.1 %] spectra) were correctly identified as *B. pseudomallei* without any misidentification, but one giving no identification. For the Australian isolates, 35/75 (46.7 %) isolates (136/685 [19.9 %] spectra) were correctly identified as *B. pseudomallei*, 7/75 (9.3 %) isolates (9/685 [1.3 %] spectra) were misidentified as *B. thailandensis*, and 33/75 (44.0 %) isolates (540/685 [78.8 %] spectra) were not identified by the SMRU SuperSpectra (Table 1).

### Identification of Asian isolates using Townsville SuperSpectra

Using seven Townsville SuperSpectra alone, 60/67 (89.6 %) Asian isolates (197/239 [82.4 %] spectra) from the MORU network laboratories were correctly identified. No identifications were obtained for 32/239 (13.4 %) spectra, and 7/67 (10.5 %) isolates (10/239 [4.2 %] spectra) were misidentified as *B. thailandensis.*

### Validation of SMRU and Townsville SuperSpectra

**Fig. 2:**
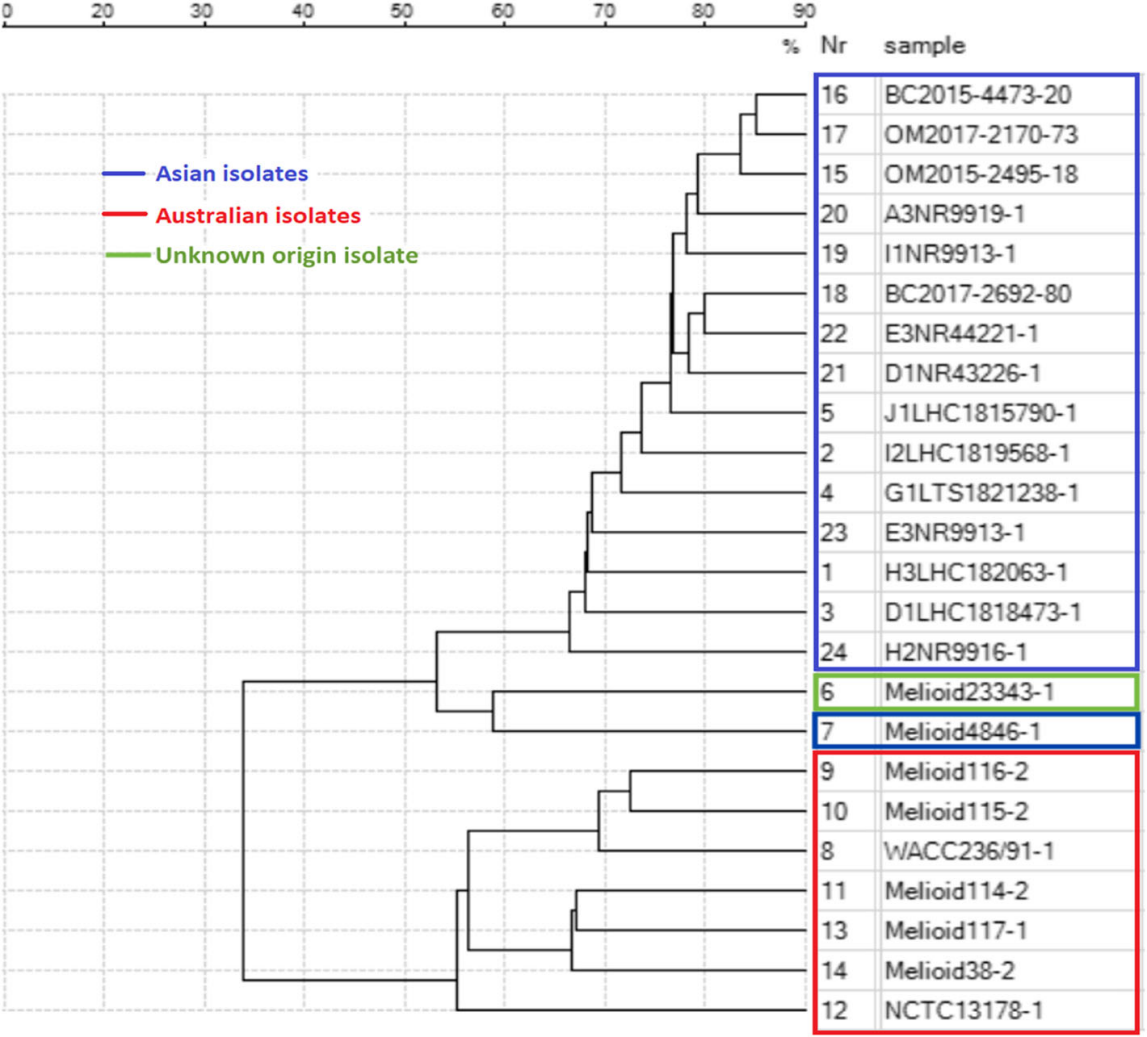
Dendrogram representing variation between geographical isolates of *Burkholderia pseudomallei* used in the study

To improve the database to be able to capture all of *B. pseudomallei* isolates from Asia and Australia, SuperSpectra SMRU-SS-Bps2 and SMRU-SS-Bps3 were created from the Townsville spectra at SMRU and activated (Fig. 1, step 5). An attempt to create a SuperSpectrum from combined SMRU and Townsville spectra was also performed, however it was not successful. During the SuperSpectrum creation, spectra variation between different geographical locations was observed (Fig. 2). When databases containing 10 *B. pseudomallei* SuperSpectra and one *B. thailandensis* SuperSpectrum (Fig. 1, step 5) were used at three different sites (SMRU, COMRU and Townsville) 100 % of the *B. pseudomallei* were correctly identified; 119 isolates from Asia and 31 isolates from Australia (Table 1). There was no misidentification of 34 non-*B. pseudomallei* isolates (292 spectra) including the closely related species, *B. thailandensis* and *B. cepacia*, none were misidentified as *B. pseudomallei* (Fig. 1, step 6).

## Discussion

This study shows the effectiveness of using the Vitek MS for the identification of *B. pseudomallei* isolates from clinical specimens. A combination of the SMRU and Townsville SuperSpectra were validated against SE Asian (*n* = 119) and Australian (*n* = 31) isolates, and correctly identified 100 % of these. In addition, none of 34 non-*B. pseudomallei* isolates (including the closely related species *B. thailandensis and B. cepacia*) were misidentified as *B. pseudomallei.*

The two previous studies using the Vitek MS mainly used isolates from one country of origin. The use of isolates from at least two different geographical areas to create these SuperSpectra allows the potential to cover diverse isolates. The initial SMRU SuperSpectrum did not have a high percentage of identification against the Australian isolates. However, when further SuperSpectra were created from the Townsville spectra and combined with the SuperSpectra that were shared from Townsville [18], a higher percentage of identification was observed. Moreover, one isolate from Lao-Oxford-Mahosot Hospital-Wellcome Trust Research Unit (LOMWRU) that was not given an identification using the initial SMRU SuperSpectrum was identified by the SuperSpectra from Townsville. The variation between different geographical isolates observed in this study (Fig. 2) could explain why the SuperSpectrum created with isolates from a limited geographical range did not capture all of the isolates. This illustrates the need to include a diverse geographical range of *B. pseudomallei* isolates for SuperSpectra creation. In addition, it was not possible to create a single SuperSpectrum that would cover all *B. pseudomallei* in this study because the variation between different geographical isolates was too diverse to create a successful one. Hence, this study ultimately resulted in 10 different SuperSpectra in the library. This is an indication of the geographical variability of the organism and also an indication of the limitations of the SuperSpectrum creation algorithm. A literature search has shown that variability in spectra of different strains of the same bacterial species has previously been observed and used for typing purposes [23, 24], but did not reveal any descriptions of the need to include strains from different geographical areas for identification purposes.

The routine direct spotting method was chosen in this study because it is a simple and cost-effective approach, and in some laboratories may be used when *B. pseudomallei* is not suspected. A previous study showed that routine thin layer application of organism to a steel Bruker MALDI-TOF plate overlaid with matrix inactivated the bacteria, however growth occurred from sub-cultured spots when a heavier amount of culture was used [25]. In our study, none of the matrix-overlaid *B. pseudomallei* spots grew on the sub-cultured plates when viability checks were performed, which provides some reassurance from a safety perspective. However, it is imperative that standard safety precautions are taken when using this method including carrying out all of the work in a biosafety cabinet with appropriate personal protective equipment (PPE); and ensuring the correct quantity of sample is added to the slide with the spot overlaid with matrix immediately.

The two previous studies that evaluated the use of the Vitek MS for the identification of *B. pseudomallei* used an extraction step before adding sample to the Vitek MS slide [18, 19]. This extraction step could help with the consistency of the spot quality and the safety of slide preparation when working with pathogenic bacteria. Although it would be preferable to have *B. pseudomallei* added to the IVD database, by validating the RUO database using the spot method, it lends itself to direct re-testing of spots that have previously been read in IVD mode, reducing time and workload. In addition, the finding that using three spots for each isolate improves the chances of identification agrees with the Townsville study [18].

The non-inclusion of Risk Group 3 (RG3)/Security Sensitive Biological Agents (SSBA) in the standard IVD databases for both the Vitek MS and MALDI Biotyper systems may result in mishandling of RG3 organisms. There have been several reports from America and Canada of incidents of exposure to *B. pseudomallei* in the laboratory due to misidentification by the MALDI-TOF [13, 26], but this potentially applies to other SSBAs as well. Therefore, until RG3 organisms (including strains from wide geographical origins) are included in both the MALDI Biotyper and the Vitek MS IVD databases, care should be taken for suspected isolates, and MALDI-TOF results not solely relied upon.

Using the MALDI-TOF, the time to identification of *B. pseudomallei* can be reduced to hours when performed from the culture plate colonies or can be reduced to 24 h when performed following a blood culture flagging positive, compared to up to two days using API 20NE. This is important in melioidosis endemic settings such as SE Asia where fast identification can assist patient management. Most empiric pneumonia or sepsis treatments do not cover *B. pseudomallei* because of its intrinsic resistance, unless there is a high index of clinical suspicion. Delayed treatment can result in poorer outcome.

There are several limitations to this study. While the study was carried out using isolates from four different countries, not all geographical regions where *B. pseudomallei* is endemic were covered and there is still the possibility that different strains might fail to identify using the developed SuperSpectra. There were only limited isolates used from each site which might not cover the wide distribution of strains found in each country. Further study to improve the reproducibility of the identification to a wider range of isolates is required. While misidentification remains a possibility, it is suggested that in endemic areas, suspected colonies should still be confirmed by an alternative method e.g. latex agglutination or API 20NE. There were only five reference *B. pseudomallei* isolates from environmental samples used in this study. A further evaluation of the utility of these SuperSpectra for the identification of *B. pseudomallei* from environmental samples would be useful. Different users at each site may also have had an impact on spot quality, and isolates were not tested at more than one study site. This study showed that multiple spots are required to increase chance of identification. Therefore, staff training and practice prior to use of the system is recommended. Given that our current database containing the SMRU and Townsville SuperSpectra showed promising results, the SuperSpectra from this study are available at figshare repository.

Ideally the closely-related species *Burkholderia mallei* would also have been included in this study. However, this organism is not readily available and it is a restricted agent in most countries. Fortunately, human disease is extremely rare and it can be differentiated by simple bench tests. However, consideration should be given to including *B. mallei* in future studies if possible.

## Conclusions

This is the first study to develop *B. pseudomallei* SuperSpectra created from isolates from at least three countries in two different geographical locations and validated with over 184 bacterial isolates including 150 isolates of *B. pseudomallei* using the Vitek MS. Using the SuperSpectra generated during this study, the Vitek MS can be used for rapid identification of *B. pseudomallei*, with three spots per isolate recommended.

## Methods

### Study sites

The study was conducted in three Mahidol-Oxford Tropical Medicine Research Unit (MORU) network sites where Vitek MS (bioMérieux, Marcy-l’Étoile, France) were used: the Shoklo Malaria Research Unit (SMRU), Mae Sot, Thailand; the Lao-Oxford-Mahosot Hospital-Wellcome Trust Research Unit (LOMWRU), Vientiane, Lao People’s Democratic Republic (Lao PDR); and the Cambodia Oxford Medical Research Unit (COMRU), Siem Reap, Cambodia, and Townsville hospital, Townsville, Australia. The study flowchart is shown in Fig. 1.

### Isolates

Details of the bacterial isolates used in this study are provided in Additional file 1, Table S1. In summary, isolates from MORU, SMRU, LOMWRU, COMRU and Townsville were included: 243 *B. pseudomallei*, 14 *B. thailandensis*, eight *Burkholderia cepacia*, nine non-*Burkholderia* Gram-negative bacteria and six Gram-positive bacteria. *B. pseudomallei* clinical isolates were from SMRU clinic catchment areas on the Thailand-Myanmar border, Tak province; Central and Southern Lao PDR; Siem Reap province, Cambodia and Townsville, Australia.

### Conventional identification of *B. pseudomallei* isolates

All *B. pseudomallei* isolates were identified using various conventional techniques including: culture and morphology on different media types, Gram staining, oxidase test, *B. pseudomallei* latex agglutination test, biochemical tests and the three disc antibiotic test that includes colistin, gentamicin and amoxicillin/clavulanic acid.

### Viability check

To investigate the safety of the slide preparation method in biosafety level 2 (BSL2) laboratories, a viability check following the routine direct spotting method was carried out at SMRU, LOMWRU and COMRU. In a biological safety cabinet, a single *B. pseudomallei* colony was taken and smeared onto a target spot on a Vitek MS slide (bioMérieux). 1 µl of α-Cyano-4-hydroxycinnamic acid (CHCA) matrix (bioMérieux) was added to the spot and allowed to dry. When fully dried, a sterile cotton swab was used to scrape the spot containing the organism and matrix and was inoculated onto a blood agar plate. The plate was then incubated at 37 °C aerobically for 14 days and checked daily for growth. This was performed in triplicate at each site.

### Vitek MS spectra acquisition

Using the routine direct spotting method, a single colony of bacterial isolate was smeared onto the target spot on the Vitek MS slide. 1 µL of CHCA matrix was then added to the spot and allowed to dry before being loaded into the Vitek MS. Spectrum acquisition was performed in the RUO mode using the Shimadzu Biotech Launchpad MALDI-TOF MS application (Shimadzu Biotech, Kyoto, Japan). The spectra were obtained within a mass range of 2,000 to 20,000 Da. *Escherichia coli* ATCC 8739 was used as a control calibration spot for each daily run following the manufacturer’s instruction (bioMérieux). The data were then transferred and stored on the SARAMIS server (bioMérieux).

### SuperSpectra creation and initial testing

At SMRU, two SuperSpectra were initially created: one for *B. pseudomallei* referred to as SMRU-SS-Bps1 and one for *B. thailandensis* referred to as SMRU-SS-Bth1. SMRU-SS-Bps1 was made using 17 reference isolates of *B. pseudomallei* and SMRU-SS-Bth1 using three reference isolates of *B. thailandensis* (Additional file 1, Table S1) which were spotted in triplicate and quadruplicate respectively for reference spectra acquisition and SuperSpectra creation. SuperSpectra creation was performed using the SARAMIS Premium application, version 4.15 (bioMérieux, Additional file 1, Method S1). The *B. pseudomallei* SuperSpectrum, SMRU-SS-Bps1, was created from 46 spectra (17 reference isolates) and the *B. thailandensis* SuperSpectrum, SMRU-SS-Bth1, was created from 12 spectra (three reference isolates) to improve the specificity of identification with any overlapping mass peaks from the *B. pseudomallei* SuperSpectrum.

The SuperSpectra were then tested using the 17 reference (MORU) and five clinical (SMRU) isolates of *B. pseudomallei*, and the three reference isolates of *B. thailandensis* from MORU (Additional file 1, Table S1). All 25 isolates were tested in triplicate.

### Validation of the SMRU SuperSpectra

At LOMWRU, 19 clinical isolates of *B. pseudomallei* were spotted in triplicate, three reference isolates of *B. thailandensis* and five clinical isolates of *B. cepacia* were spotted in duplicate (Additional file 1, Table S1). The spectra were acquired and compared with the LOMWRU RUO database including the imported and activated SMRU SuperSpectra, SMRU-SS-Bps1 and SMRU-SS-Bth1. At COMRU, 30 clinical isolates of *B. pseudomallei* from Cambodia were spotted in triplicate. The spectra were acquired and sent to SMRU for further analysis.

At SMRU, five clinical isolates of *B. pseudomallei* and 15 non-*Burkholderia* bacteria were spotted in triplicate. The spectra were acquired and compared with the SMRU database containing the SMRU SuperSpectra, SMRU-SS-Bps1 and SMRU-SS-Bth1. In addition, the spectra from COMRU and 715 existing spectra acquired from 76 isolates of *B. pseudomallei* from the Townsville study were also analysed using the SMRU database containing SMRU-SS-Bps1 and SMRU-SS-Bth1 SuperSpectra.

### Additional SuperSpectra creation

Following the same method (Additional file 1, Method S1), two additional *B. pseudomallei* SuperSpectra, SMRU-SS-Bps2 and SMRU-SS-Bps3, were created at SMRU from 100 spectra (four reference isolates) and 330 spectra (34 reference and clinical isolates), respectively from the shared Townsville spectra. In addition, seven *B. pseudomallei* SuperSpectra from the Townsville study were shared and included in the SMRU database (Townsville-SS-Bps128, Townsville-SS-Bps347, Townsville-SS-Bps457, Townsville-SS-Bps694, Townsville-SS-Bps854, Townsville-SS-BpsATCC4846, and Townsville-SS-BpsATCC23343). Masses of all SuperSpectra used in this study are shown in Additional file 1, Table S2. The SuperSpectra are deposited at the figshare repository for open access and Digital Object Identifier (DOI) is 10.6084/m9.figshare.13359389 (doi.org).

### Validation of SMRU and Townsville SuperSpectra

All SMRU (SMRU-SS-Bps1 to 3, and SMRU-SS-Bth1) and seven Townsville *B. pseudomallei* SuperSpectra were activated and validated with 184 clinical and reference isolates that were not used in the SuperSpectra creations at three different study sites (SMRU, COMRU and Townsville): *B. pseudomallei* (357 spectra acquired from 119 Asian isolates, and 62 spectra acquired from 31 Australian isolates) and non-*B. pseudomallei* isolates (292 spectra acquired from 15 non-*Burkholderia* species, 11 isolates of *B. thailandensis*, and eight isolates of *B. cepacia*).

## Supplementary Information

**Additional file 1: Table S1. **Bacterial isolates used in this study. **Table S2. **Masses of the SuperSpectra used in this study. **Figure S1. **Spot images on Vitek MS slide (bioMérieux) by Shimadzu Biotech Launchpad application showing quality of slide preparation: 1A1 to 2A3 and 2D1 to 2G3 = spots of *B. pseudomallei*, 2A4 to 2C4 = spots of *B. thailandensis*, and 2G4 = spot of *Escherichia coli *prepared at SMRU; * = spot with no identification. **Method S1. **SuperSpectra creation using SARAMIS Premium application, version 4.15 (bioMérieux).

## Data Availability

The SuperSpectra generated and/or analysed during the current study are available in the figshare repository, 10.6084/m9.figshare.13359389 (doi.org).

## Abbreviations

BSL2: Biosafety level 2;
CHCA: α-Cyano-4-hydroxycinnamic acid
COMRU: Cambodia Oxford Medical Research Unit
DOI: Digital object identifier
FDA: Food and drug administration
IVD: *In-vitro* diagnostic
Lao PDR: Lao People’s Democratic Republic
LOMWRU: Lao-Oxford-Mahosot Hospital-Wellcome Trust Research Unit
MLADI-TOF MS: Matrix assisted laser desorption/ionization time-of-flight mass spectrometry
MORU: Mahidol-Oxford Tropical Medicine Research Unit
PPE: Personal protective equipment
RG3: Risk group 3
RUO: Research use only
SE Asia: South-East Asia
SMRU: Shoklo Malaria Research Unit
SSBA: Security sensitive biological agents
