# Supplementary material for: A multi-country study using MALDI-TOF mass spectrometry for rapid identification of Burkholderia pseudomallei
Source: BMC Microbiol. 2021 Jul 16;21:213. doi: 10.1186/s12866-021-02276-1 (PMC8283998; doi:10.1186/s12866-021-02276-1)
Supplement: Supplementary file 1 — Additional file 1: Table S1. Bacterial isolates used in this study. Table S2. Masses of the SuperSpectra used in this study. Figure S1. Spot images on Vitek MS slide (bioMérieux) by Shimadzu Biotech Launchpad application showing quality of slide preparation: 1A1 to 2A3 and 2D1 to 2G3 = spots of B. pseudomallei, 2A4 to 2C4 = spots of B. thailandensis, and 2G4 = spot of Escherichia coli prepared at SMRU; * = spot with no identification. Method S1. SuperSpectra creation using SARAMIS Premium application, version 4.15 (bioMérieux). [file 12866_2021_2276_MOESM1_ESM.docx]

**A multi-country study using MALDI-TOF mass spectrometry for rapid identification of *Burkholderia pseudomallei***

Wanitda Watthanaworawit^1*^, Tamalee Roberts^2^, Jill Hopkins^3,4^, Ian Gassiep^5^, Robert Norton^6,7^, Matthew T. Robinson^2,4^, Joy Silisouk^2^, Poda Sar^3^,Sena Sao^3^, Premjit Amornchai^8^, Direk Limmathurotsakul^8^, Vanaporn Wuthiekanun^8^, Francois Nosten^1,4^, Andrew J. H. Simpson^2,4^, Paul Turner^3,4^, Clare L. Ling^1,4^

^1^Shoklo Malaria Research Unit, Mahidol-Oxford Tropical Medicine Research Unit, Faculty of Tropical Medicine, Mahidol University, Mae Sot, Thailand

^2^Lao-Oxford-Mahosot Hospital-Wellcome Trust Research Unit, Microbiology Laboratory, Mahosot Hospital, Vientiane, Lao People’s Democratic Republic

^3^Cambodia Oxford Medical Research Unit, Angkor Hospital for Children, Siem Reap, Cambodia

^4^Centre for Tropical Medicine and Global Health, Nuffield Department of Medicine, University of Oxford, Oxford, United Kingdom

^5^University of Queensland Centre for Clinical Research, Herston, Queensland, Australia

^6^Pathology, Townsville Hospital, Townsville, Queensland, Australia

^7^Faculty of Medicine, University of Queensland, Brisbane, Australia

^8^Mahidol-Oxford Tropical Medicine Research Unit, Faculty of Tropical Medicine, Mahidol University, Bangkok, Thailand

*Address correspondence to Wanitda Watthanaworawit, [wanitda@tropmedres.ac](mailto:wanitda@tropmedres.ac)

**Supplementary information**

**File name: Additional file 1.**

**File format: .docx**

**Title and description of data:**

**Table S1** Bacterial isolates used in this study;

**Table S2** Masses of the SuperSpectra used in this study;

**Fig. S1** Spot images on Vitek MS slide (bioMérieux) by Shimadzu Biotech Launchpad application showing quality of slide preparation: 1A1 to 2A3 and 2D1 to 2G3 = spots of *B. pseudomallei*, 2A4 to 2C4 = spots of *B. thailandensis*, and 2G4 = spot of *Escherichia coli* prepared at SMRU; * = spot with no identification;

**Method S1** SuperSpectra creation using SARAMIS Premium application, version 4.15 (bioMérieux).

**Supplementary material**

**Table S1** Bacterial isolates used in this study.

| **Bacterial isolates (Total number)** | **Reference no.** | **Source** | **Origin** | **Study site collection (ref)** | **Purpose*** |
| --- | --- | --- | --- | --- | --- |
| *Burkholderia pseudomallei* (243) | NR-9910 | Human blood | Thailand | MORU | A, C |
|  | NR-9911 | Human blood | Thailand | MORU | A, C |
|  | NR-9912 | Human blood | Thailand | MORU | A, C |
|  | NR-9913 | Human blood | Thailand | MORU | A, C |
|  | NR-9914 | Human pus | Thailand | MORU | A, C |
|  | NR-9915 | Human swab from non-sterile site | Thailand | MORU | A, C |
|  | NR-9916 | Human blood | Thailand | MORU | A, C |
|  | NR-9917 | Soil | Thailand | MORU | A, C |
|  | NR-9918 | Soil | Thailand | MORU | A, C |
|  | NR-9919 | Soil | Thailand | MORU | A, C |
|  | NR-9920 | Water | Australia | MORU | A, C |
|  | NR-9921 | Human skin | Australia | MORU | A, C |
|  | NR-9922 | Human blood | Australia | MORU | A, C |
|  | NR-9923 | Human blood | Australia | MORU | A, C |
|  | NR-43226 | Soil | Thailand | MORU | A, C |
|  | NR-44220 | Human tracheal suction | Thailand | MORU | A, C |
|  | NR-44221 | Human tracheal suction | Thailand | MORU | A, C |
|  | ATCC 23343 | Human | Unknown | Townsville (18) | D, E, G |
|  | NCTC 4846 | Monkey | Singapore | Townsville (18) | D, E, G |
|  | NCTC 13178 | Brain | Australia | Townsville (18) | D, E, G |
|  | WACC 236/91 | No clinical data available | Australia | Townsville (18) | D, E, G |
|  | - | 5 Clinical isolates | Thailand-Myanmar border | SMRU | C, G, H |
|  | - | 19 Clinical isolates | Lao PDR | LOMWRU | F, H |
|  | - | 95 Clinical isolates | Cambodia | COMRU | G, H |
|  | - | 72 Clinical isolates | Australia | Townsville (18) | E, G |
|  | - | 31 Clinical isolates | Australia | Townsville | H |

| **Bacterial species (Total number)** | **Reference no.** | **Source** | **Origin** | **Study site collection (ref)** | **Purpose*** |
| --- | --- | --- | --- | --- | --- |
| *Burkholderia thailandensis* (14) | NR-9907 | Soil | Thailand | MORU | B, C |
|  | NR-9908 | Soil | Thailand | MORU | B, C |
|  | NR-9909 | Soil | Thailand | MORU | B, C |
|  | ATCC 700388 | Soil | Thailand | LOMWRU | F, H |
|  | E421 | Soil | Thailand | LOMWRU | F, H |
|  | E305 | Soil | Thailand | LOMWRU | F, H |
|  | - | 8 Clinical isolates | Australia | Townsville (18) | H |
| *Burkholderia cepacia* (8) | - | 5 Clinical isolates | Lao PDR | LOMWRU | F, H |
|  | - | 3 Clinical isolates | Cambodia | COMRU | H |
| Non-*Burkholderia* (15) |  |  |  |  |  |
| *Enterococcus faecalis* | ATCC 29212 | Urine | Portland, USA | SMRU | G, H |
| *Escherichia coli* | ATCC 25922 | Clinical isolate | Seattle, USA | SMRU | G, H |
| *Escherichia coli* | ATCC 35218 | Canine | Tennessee, USA | SMRU | G, H |
| *Haemophilus influenzae* | ATCC 49247 | Human clinical specimen – expectorated sputum | Massachusetts, USA | SMRU | G, H |
| *Pseudomonas aeruginosa* | ATCC 27853 | Blood culture | Boston, USA | SMRU | G, H |
| *Staphylococcus aureus* | ATCC 25923 | Clinical isolate | Seattle, USA | SMRU | G, H |
| *Streptococcus pneumoniae* | ATCC 49619 | Human sputum | Arizona, USA | SMRU | G, H |
| *Enterobacter cloacae* | - | 1 Clinical isolate | Thailand-Myanmar border | SMRU | G, H |
| *Haemophilus influenzae* | - | 1 Clinical isolate | Thailand-Myanmar border | SMRU | G, H |
| *Klebsiella pneumoniae* | - | 1 Clinical isolate | Thailand-Myanmar border | SMRU | G, H |
| *Pseudomonas stutzeri* | - | 1 Clinical isolate | Thailand-Myanmar border | SMRU | G, H |
| *Salmonella* Typhi | - | 1 Clinical isolate | Thailand-Myanmar border | SMRU | G, H |
| *Staphylococcus saprophyticus* | - | 1 Clinical isolate | Thailand-Myanmar border | SMRU | G, H |
| Staphylococcus coagulase negative | - | 1 Clinical isolate | Thailand-Myanmar border | SMRU | G, H |
| Streptococcus Group B | - | 1 Clinical isolate | Thailand-Myanmar border | SMRU | G, H |

*Purpose of the isolates used (A= isolates used to create SMRU-SS-Bps1, B= isolates used to create SMRU-SS-Bth1, C= isolates used to test initial SuperSpectra: SMRU-SS-Bps1 and SMRU-SS-Bth1, D= isolates used to create SMRU-SS-Bps2, E= isolates used to create SMRU-SS-Bps3, F= isolates used to validate SMRU-SS-Bps1 and SMRU-SS-Bth1 at LOMWRU, G= isolates used to validate SMRU-SS-Bps1 and SMRU-SS-Bth1 at SMRU, H= isolates used to validate all SMRU (SMRU-SS-Bps1 to 3, and SMRU-SS-Bth1) and Townsville (Townsville-SS-Bps128, Townsville-SS-Bps347, Townsville-SS-Bps457, Townsville-SS-Bps694, Townsville-SS-Bps854, Townsville-SS-BpsATCC4846, and Townsville-SS-BpsATCC23343) SuperSpectra).

**Table S2** Masses of the SuperSpectra used in this study.

| **SMRU-SS-Bps1** | **SMRU-SS-Bth1** | **SMRU-SS-Bps2** | **SMRU-SS-Bps3** | **Townsville-SS-Bps128** | **Townsville-SS-Bps347** | **Townsville-SS-Bps457** | **Townsville-SS-Bps694** | **Townsville-SS-Bps854** | **Townsville-SS-BpsATCC4846** | **Townsville-SS-BpsATCC23343** |
| --- | --- | --- | --- | --- | --- | --- | --- | --- | --- | --- |
| 3112.1 | 3112.0 | 3115.5 | 3634.2 | 3113.6 | 3112.8 | 3113.4 | 3275.9 | 3112.6 | 3256.0 | 3635.3 |
| 3244.7 | 3205.7 | 3260.1 | 4389.9 | 3114.1 | 3262.9 | 3263.2 | 4407.2 | 3263.9 | 3635.4 | 4294.9 |
| 3275.4 | 3237.1 | 3274.1 | 4405.7 | 3276.8 | 3274.7 | 3274.2 | 4812.8 | 3274.1 | 3756.7 | 4295.3 |
| 3584.5 | 3259.8 | 3274.9 | 4803.5 | 3584.3 | 3653.6 | 3654.7 | 4856.3 | 3275.8 | 4839.7 | 4408.2 |
| 3655.0 | 3323.3 | 3275.2 | 4839.3 | 3586.2 | 4409.2 | 4408.2 | 5194.3 | 3654.2 | 5178.2 | 4838.9 |
| 3725.5 | 3584.0 | 3654.7 | 5177.2 | 3655.3 | 4810.7 | 4810.2 | 5304.9 | 3776.7 | 5194.8 | 4969.2 |
| 3775.7 | 3654.1 | 3655.0 | 5192.7 | 3776.9 | 4855.7 | 4856.0 | 5791.0 | 4407.0 | 5792.8 | 4995.3 |
| 4115.7 | 3706.3 | 3655.2 | 5223.9 | 4410.8 | 4986.4 | 4986.6 | 6220.5 | 4409.5 | 6224.8 | 5009.6 |
| 4314.9 | 3755.7 | 4216.7 | 6222.5 | 4638.2 | 5194.1 | 5193.5 | 6547.4 | 4807.8 | 6532.4 | 5023.4 |
| 4410.3 | 3991.4 | 4405.2 | 6476.1 | 4660.2 | 5303.4 | 5303.5 | 6548.5 | 4809.2 | 6551.0 | 5037.6 |
| 4429.5 | 4041.9 | 4805.2 | 6518.3 | 4811.4 | 5790.3 | 5789.7 | 7034.7 | 4811.0 | 7546.9 | 5051.4 |
| 4459.1 | 4115.7 | 4814.9 | 6536.9 | 4829.5 | 6225.0 | 6223.3 | 7164.7 | 4855.3 | 7986.8 | 5065.9 |
| 4811.0 | 4275.7 | 4983.8 | 6551.4 | 4839.7 | 6238.9 | 6237.8 | 7266.6 | 4986.9 | 8111.6 | 5079.4 |
| 4858.8 | 4314.8 | 5150.2 | 7155.9 | 4854.8 | 6487.6 | 6487.7 | 7305.2 | 5150.2 | 8224.7 | 5193.8 |
| 4988.3 | 4409.8 | 5151.1 | 7165.9 | 4861.7 | 6525.8 | 6525.2 | 7308.9 | 5193.5 | 8418.4 | 5223.5 |
| 5196.2 | 4426.9 | 5431.3 | 7258.2 | 4988.7 | 6548.7 | 6547.7 | 8116.7 | 5302.6 | 8437.4 | 5415.0 |
| 5216.2 | 4458.9 | 5992.7 | 7299.2 | 5156.6 | 6660.7 | 6659.9 | 8118.1 | 5789.6 | 8447.6 | 5415.0 |
| 5216.9 | 4810.6 | 6489.8 | 7541.9 | 5196.6 | 7035.4 | 7033.7 | 8422.9 | 5809.7 | 8620.1 | 6223.7 |
| 5793.5 | 4858.4 | 6522.1 | 7984.3 | 5523.9 | 7166.6 | 7167.1 | 8438.8 | 6221.8 | 8624.0 | 6490.6 |
| 6226.3 | 4987.7 | 6527.0 | 8108.5 | 5543.0 | 7265.5 | 7265.7 | 8623.9 | 6224.0 | 8692.8 | 6535.1 |
| 6245.7 | 5195.6 | 6552.0 | 8114.7 | 5792.3 | 7307.4 | 7306.4 | 8912.9 | 6525.4 | 8912.1 | 6550.4 |
| 6265.8 | 5760.7 | 6656.6 | 8223.1 | 5986.4 | 7548.8 | 7546.1 | 9615.8 | 6548.3 | 9452.7 | 7544.9 |
| 6491.6 | 6225.9 | 7027.6 | 8354.3 | 6225.6 | 7985.5 | 8118.3 | 9617.8 | 6660.5 | 9613.6 | 7631.7 |
| 6553.1 | 6244.8 | 7160.8 | 8442.9 | 6372.2 | 8117.0 | 8227.4 | 9973.1 | 7034.6 | 9708.4 | 7984.3 |
| 6573.3 | 6490.7 | 7308.8 | 8621.5 | 6550.4 | 8229.1 | 8424.8 | 10477.9 | 7166.4 | 9968.4 | 8113.4 |
| 6592.0 | 6522.7 | 7860.4 | 8693.6 | 6552.3 | 8358.3 | 8444.1 | 10485.2 | 7168.0 | 10479.1 | 8221.7 |
| 7169.7 | 6931.4 | 8224.0 | 8910.7 | 6555.6 | 8425.6 | 8623.3 | 10603.1 | 7307.6 | 10603.9 | 8465.0 |
| 7191.7 | 7169.4 | 8694.7 | 9453.2 | 7169.4 | 8444.2 | 8918.0 | 10858.5 | 7546.4 | 10858.2 | 8479.9 |
| 7311.8 | 7310.9 | 8698.5 | 9613.6 | 7311.5 | 8622.6 | 9617.3 | 11509.6 | 7552.6 | 11097.4 | 8491.4 |
| **SMRU-SS-Bps1** | **SMRU-SS-Bth1** | **SMRU-SS-Bps2** | **SMRU-SS-Bps3** | **Townsville-SS-Bps128** | **Townsville-SS-Bps347** | **Townsville-SS-Bps457** | **Townsville-SS-Bps694** | **Townsville-SS-Bps854** | **Townsville-SS-BpsATCC4846** | **Townsville-SS-BpsATCC23343** |
| 7553.5 | 7415.6 | 8921.3 | 9709.7 | 7553.8 | 8628.6 | 9970.5 | 11992.6 | 7985.8 | 11125.1 | 8498.6 |
| 7593.9 | 7923.6 | 10700.8 | 9967.8 | 7909.9 | 8698.0 | 10483.0 | 15272.5 | 8119.1 | 11162.5 | 8517.9 |
| 8122.3 | 7986.5 | 10832.7 | 10479.3 | 8123.4 | 8914.9 | 10605.6 | 15276.5 | 8229.2 | 11515.6 | 8619.7 |
| 8263.5 | 8086.7 | 10839.3 | 10603.4 | 8230.1 | 9618.1 | 10862.6 | 15297.3 | 8443.3 | 11822.0 | 8910.2 |
| 8364.8 | 8233.6 | 11125.8 | 10863.7 | 8237.4 | 9966.3 | 11095.7 | 19870.2 | 8625.4 | 11993.5 | 9450.9 |
| 8631.6 | 8553.8 | 11158.9 | 11128.2 | 8440.1 | 9973.3 | 11096.6 | 19901.0 | 9617.2 |  | 9612.4 |
| 8920.3 | 8654.0 | 11818.5 | 11514.4 | 8452.1 | 10483.8 | 11510.9 |  | 9622.7 |  | 9707.3 |
| 9621.8 | 8919.1 | 14241.4 | 11820.3 | 9622.6 | 10606.4 | 11513.6 |  | 9969.8 |  | 9967.3 |
| 9659.4 | 9621.3 | 15272.8 | 11991.1 | 9713.0 | 10703.0 | 11995.9 |  | 10482.7 |  | 10111.0 |
| 9717.6 | 9716.5 | 19843.7 | 15294.8 | 9955.8 | 10837.3 | 15276.5 |  | 10603.4 |  | 10477.7 |
| 9974.9 | 9973.9 | 19887.1 | 19882.8 | 9967.2 | 10862.6 | 15297.6 |  | 15275.5 |  | 10602.5 |
| 10490.5 | 10487.7 | 19921.9 | 19914.1 | 9976.8 | 11104.7 | 19846.7 |  | 15297.5 |  | 10859.3 |
|  |  |  |  |  | 11509.1 | 19881.8 |  |  |  | 11123.0 |
|  |  |  |  |  | 11991.7 | 19920.5 |  |  |  | 11510.5 |
|  |  |  |  |  | 15275.5 |  |  |  |  | 11822.0 |
|  |  |  |  |  | 19853.1 |  |  |  |  | 13057.9 |
|  |  |  |  |  | 19908.8 |  |  |  |  | 15294.4 |
|  |  |  |  |  |  |  |  |  |  | 19895.1 |
|  |  |  |  |  |  |  |  |  |  | 19960.2 |


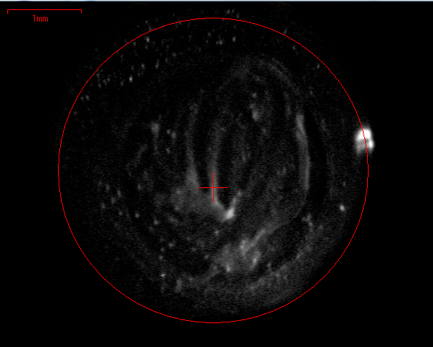

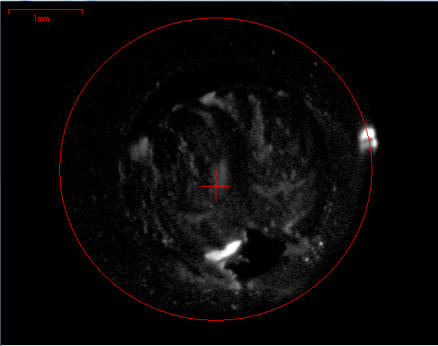

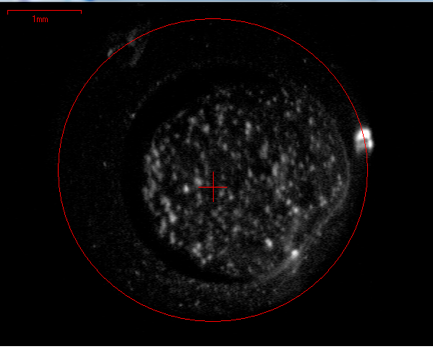

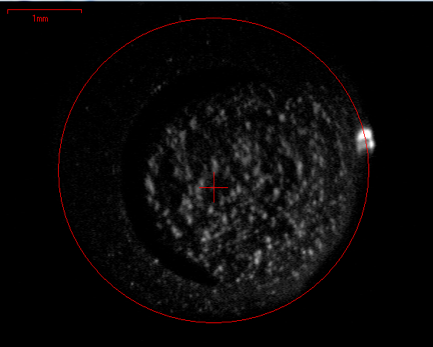


1B4

1B3

1B2

1B1


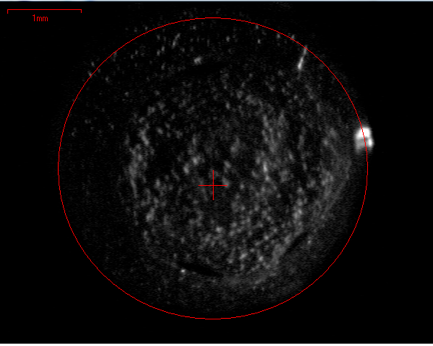

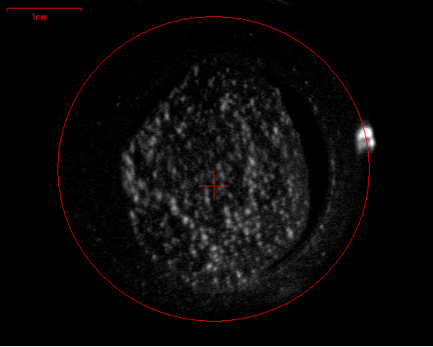

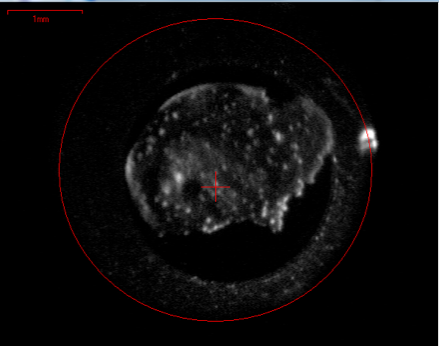

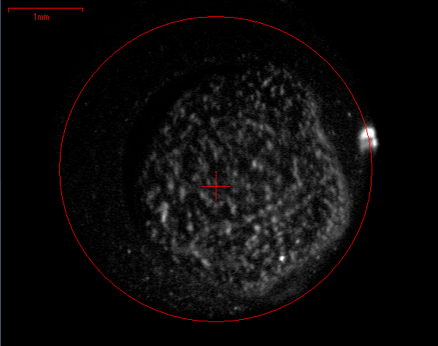


1C4

1C3

1C2

1C1


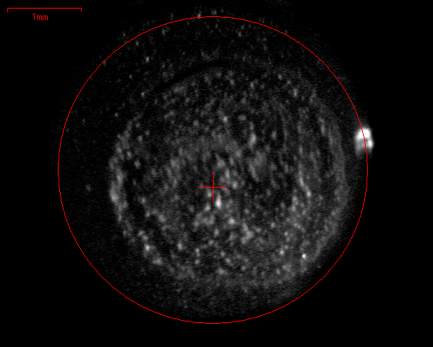

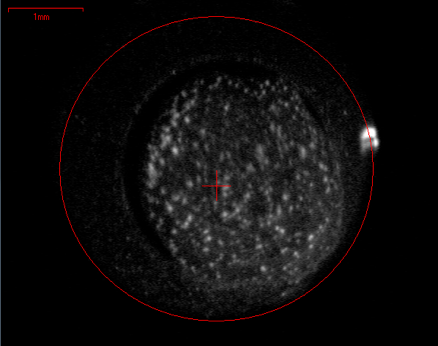

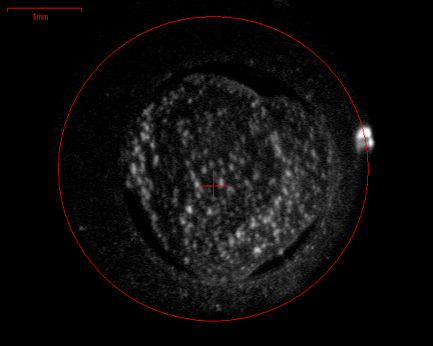

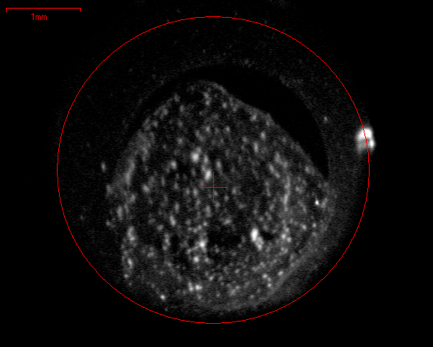


1D4

1D3

1D2

1D1


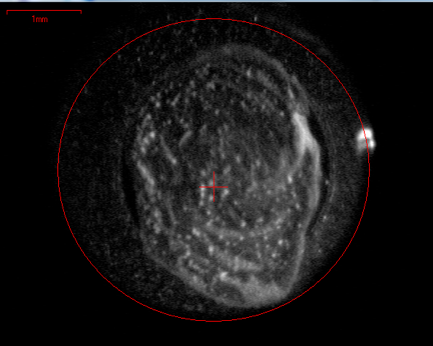

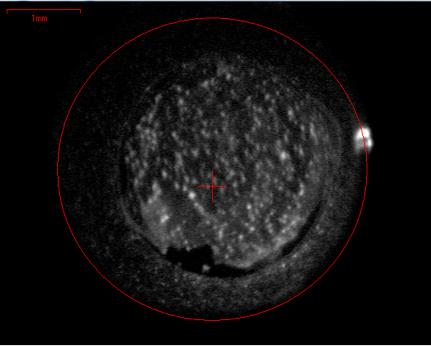

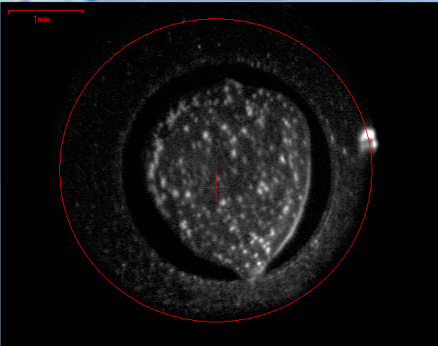

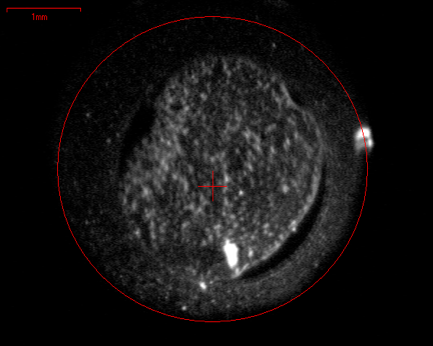


1E4*

1E3*

1E2

1E1


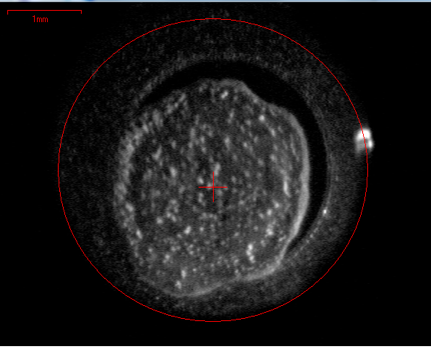

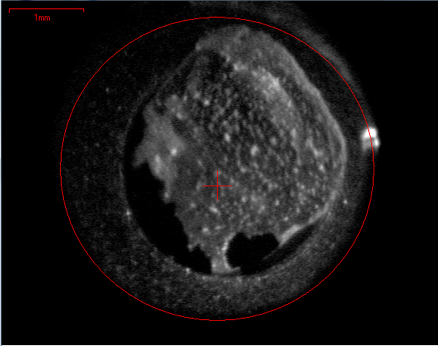

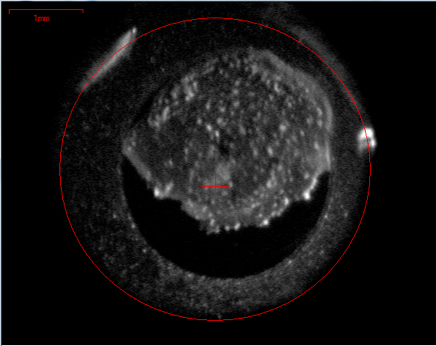

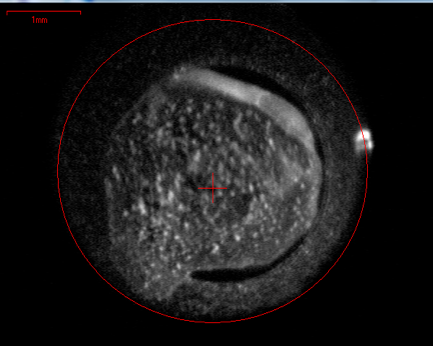


1F2*

1F4

1F3

1F1


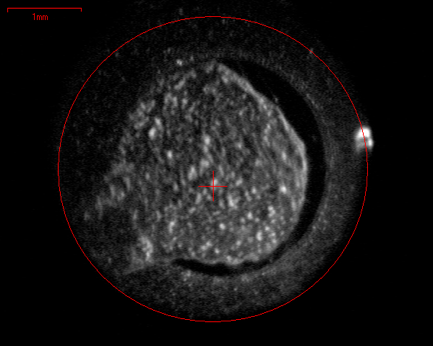

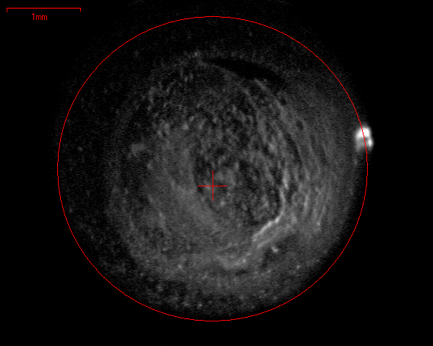

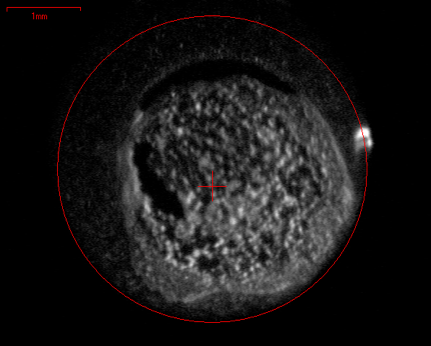

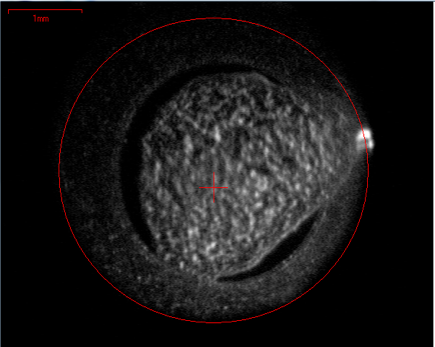


1G4*

1G3*

1G2

1G1


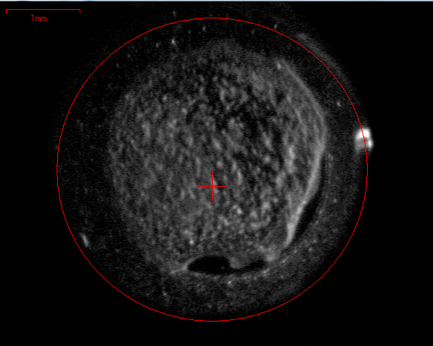

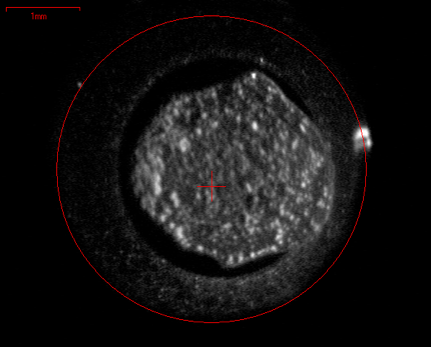

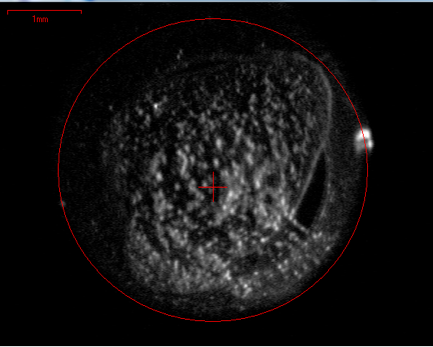

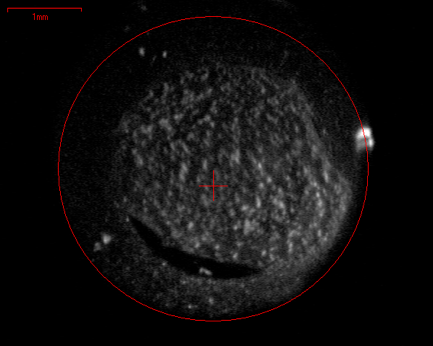


1H4*

1H3*

1H2*

1H1


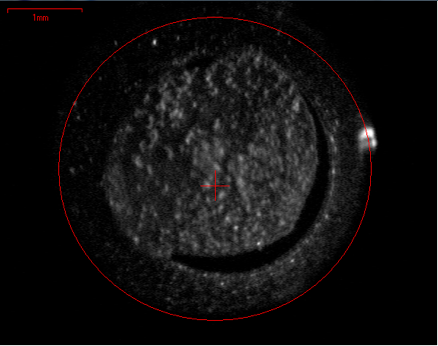

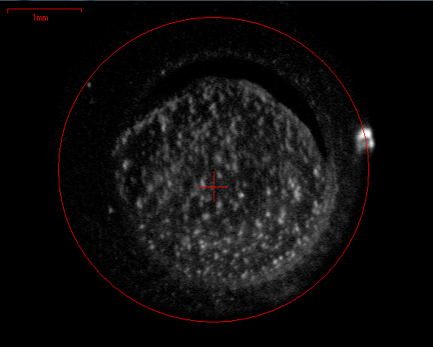

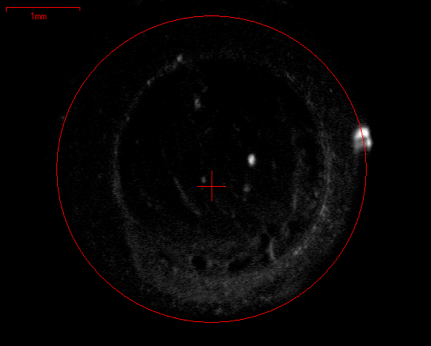

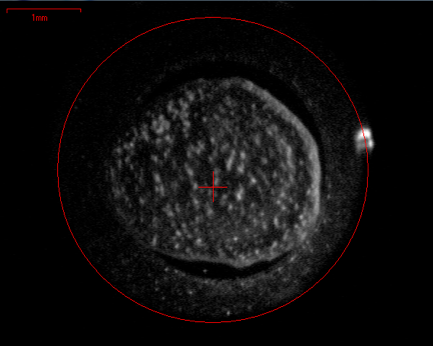


1I3*

1I4

1I2

1I1


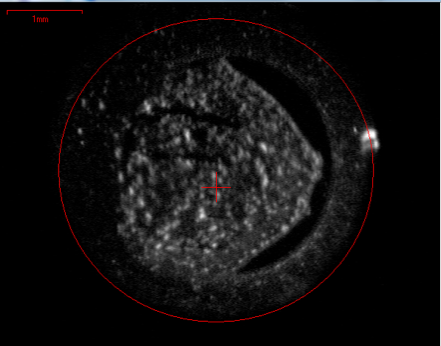

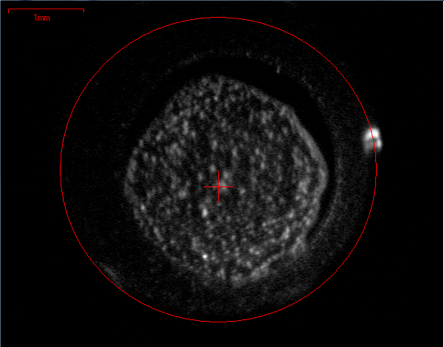

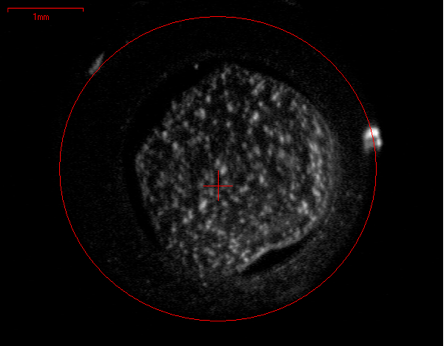

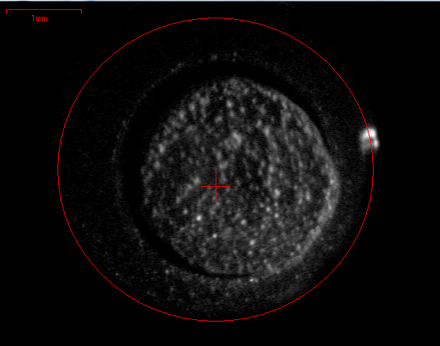


1J4

1J3

1J2

1J1


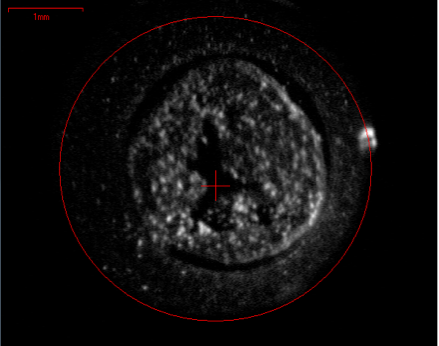

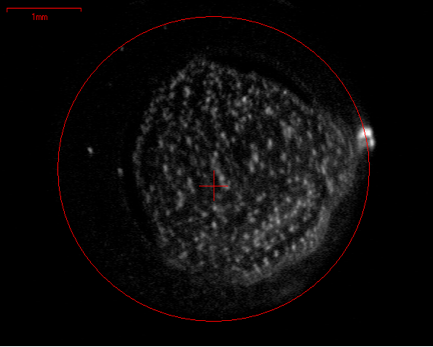

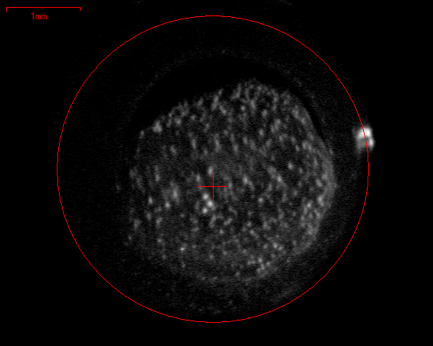

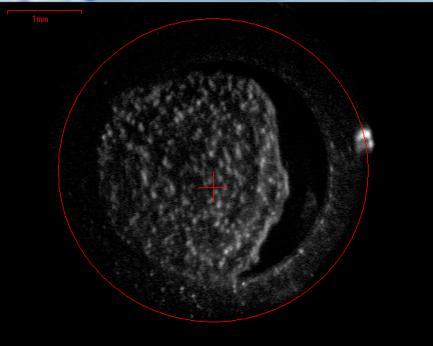


1K4

1K3

1K2

1K1


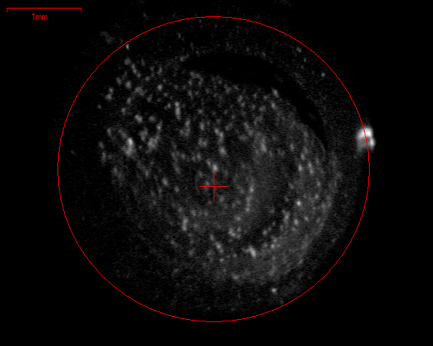

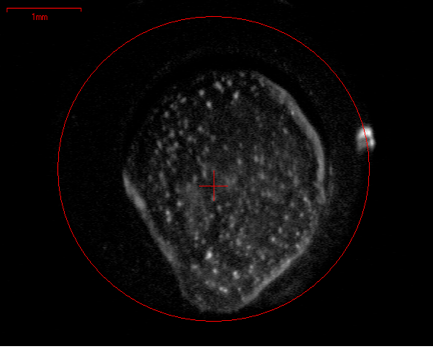

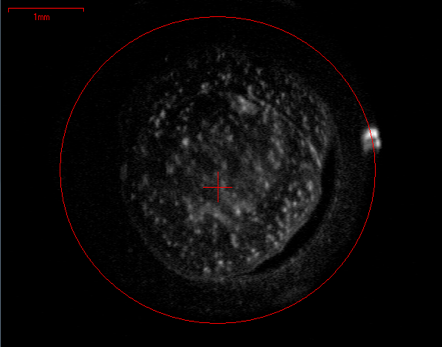

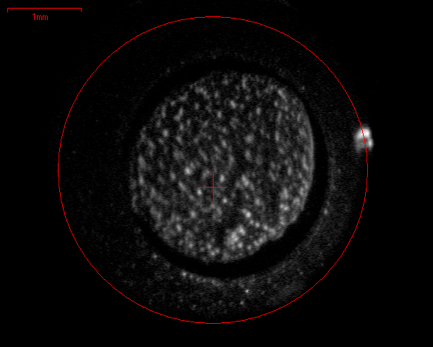


1L4

1L3

1L2

1L1


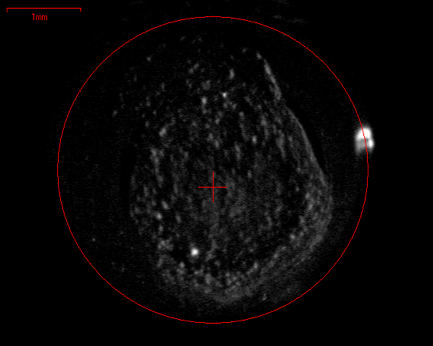

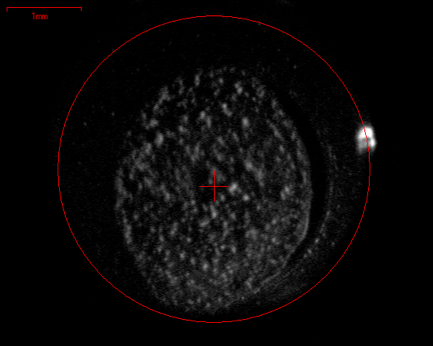

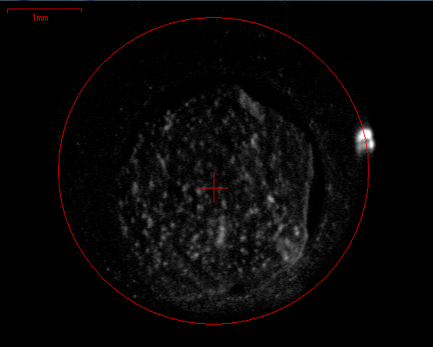

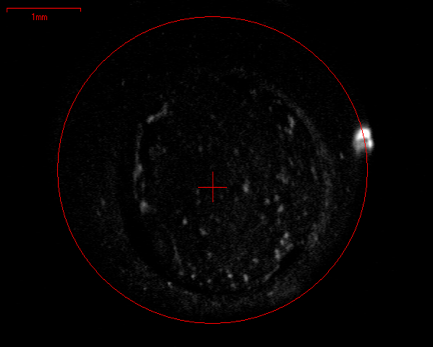


2A4

2A3

2A2

2A1


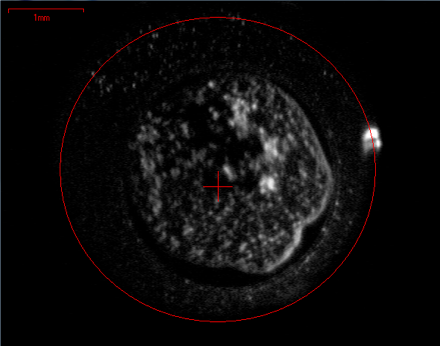

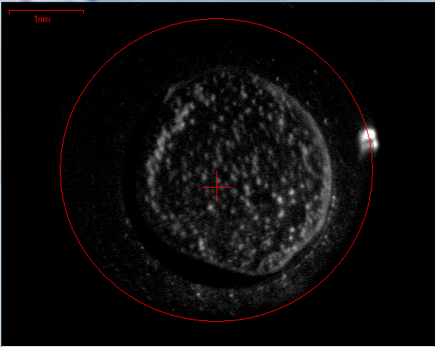

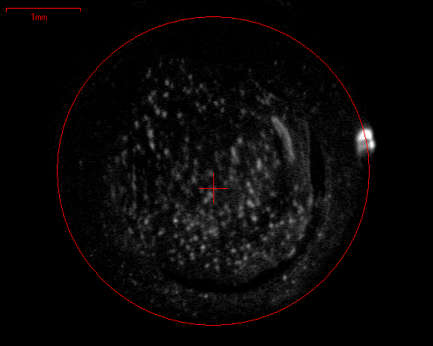

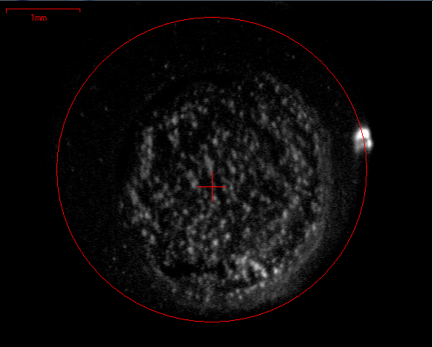


2B4

2B3

2B2

2B1


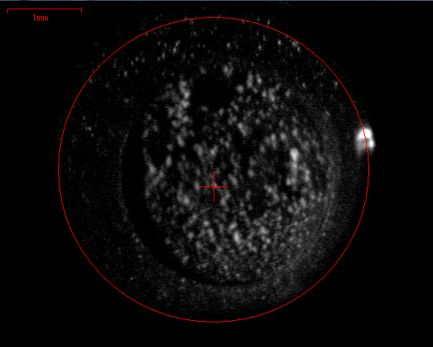

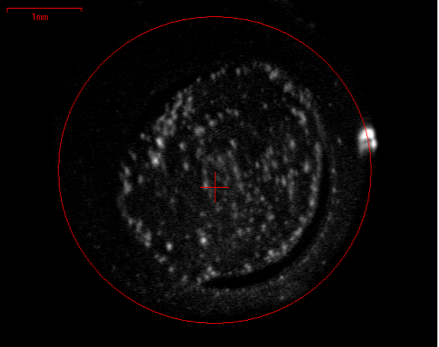

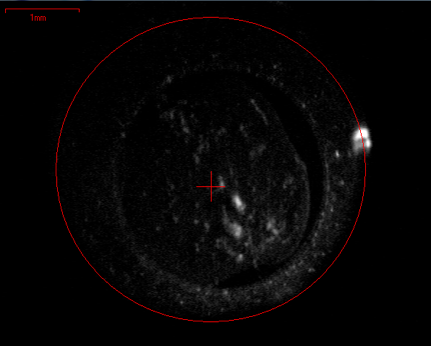

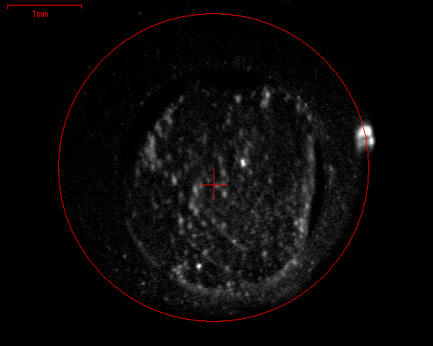


2C4

2C3

2C2

2C1


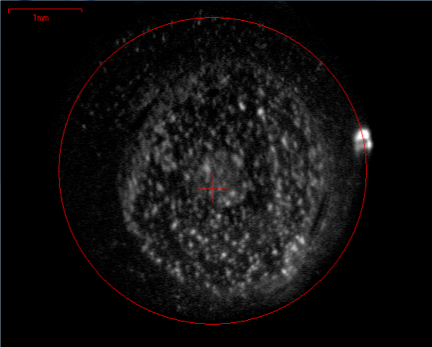

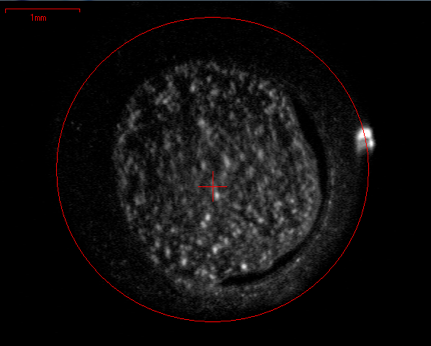

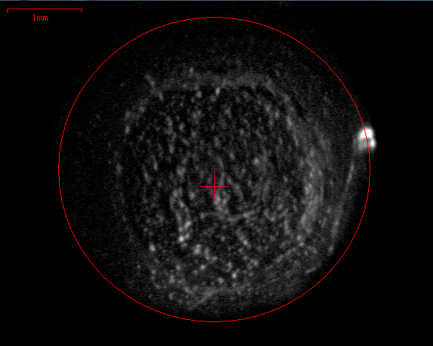

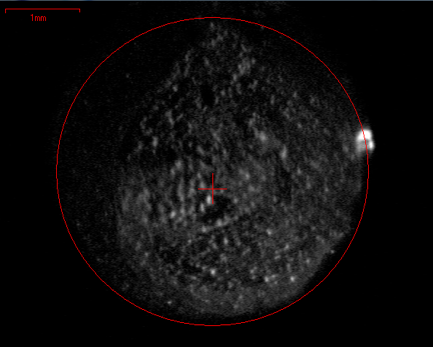


2D4

2D3

2D2

2D1


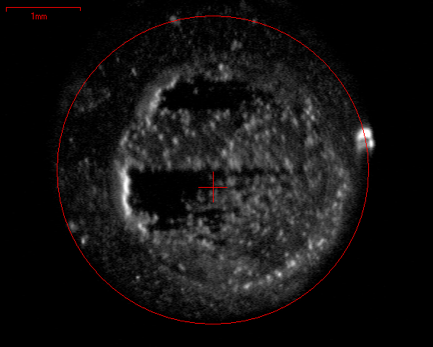

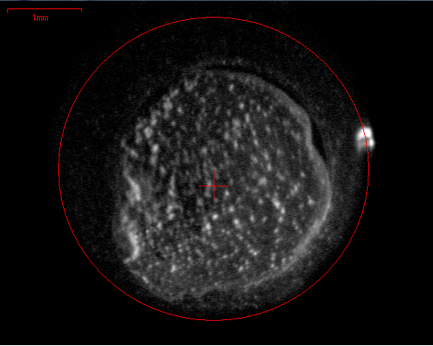

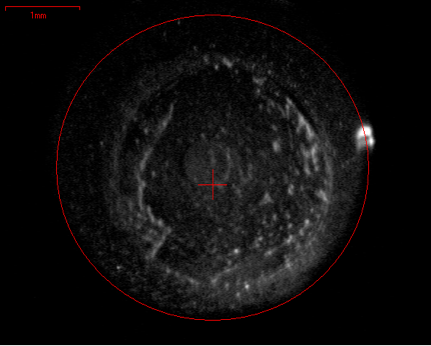

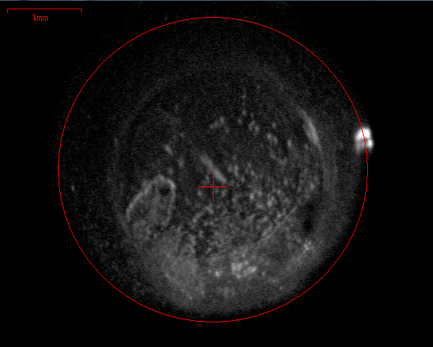


2E4

2E3

2E2

2E1


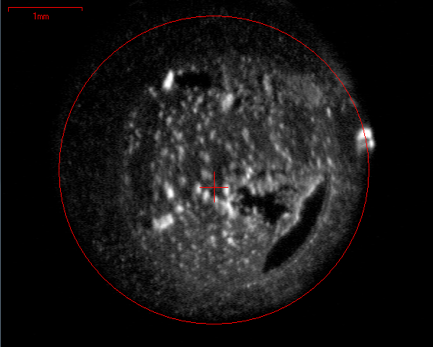

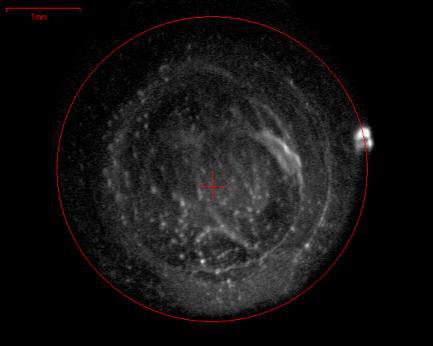

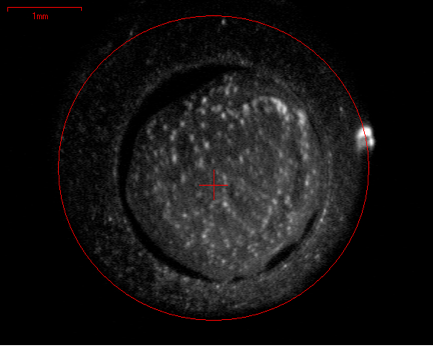

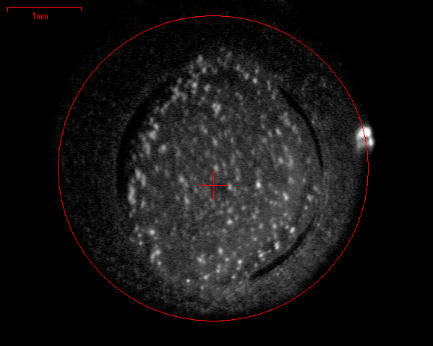


2F4

2F3

2F2

2F1


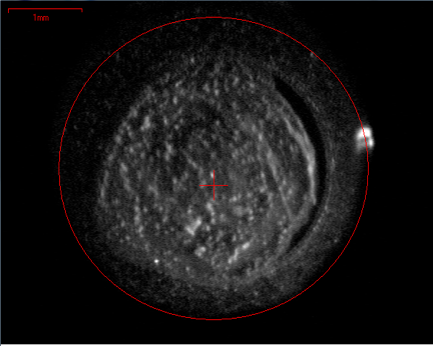

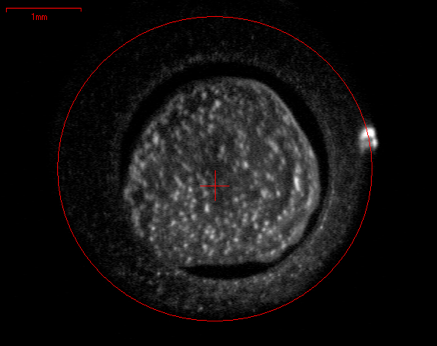

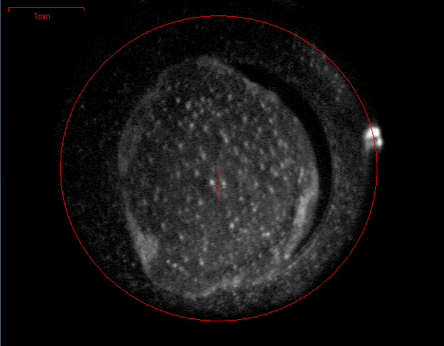

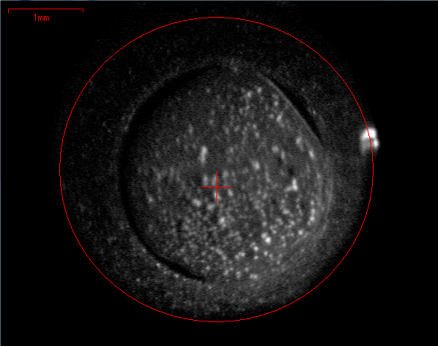


2G4

2G3

2G2

2G1

**Fig. S1** Spot images on Vitek MS slide (bioMérieux) by Shimadzu Biotech Launchpad application showing quality of slide preparation: 1A1 to 2A3 and 2D1 to 2G3 = spots of *B. pseudomallei*, 2A4 to 2C4 = spots of *B. thailandensis*, and 2G4 = spot of *Escherichia coli* prepared at SMRU; * = spot with no identification.

**Method S1** SuperSpectra creation using SARAMIS Premium application, version 4.15 (bioMérieux).

Spectra were selected if they had >80 to <250 peaks. A SuperSpectrum was created with 70% similarity between replicates and 65% similarity overall. The SuperSpectrum was then filtered to obtain a list of 39-41 specific masses. The selected spectra were compared to the whole database. The first non-target species that matched was identified to obtain the value of “matches”. The number of matched peaks of non-target species after comparison to the whole database was compared to the number of specific masses in the SuperSpectrum. The target number code (peak weight) between 28 and 35 was sought and assigned the weight found for each spectrum in the number code for the masses selected.
